# New-onset chronic inflammatory demyelinating polyradiculoneuropathy after COVID-19 infection: a case report

**DOI:** 10.1097/MS9.0000000000000352

**Published:** 2023-07-31

**Authors:** Mohammad Abedi Samakoush, Lotfollah Davoodi, Mojtaba Khademian, Saeed Kargar-soleimanabad, Mohammad-Ali Abedini

**Affiliations:** a Antimicrobial Resistance Research Center; b Antimicrobial Resistance Research Center, Communicable Diseases Institute, and Department of Infectious Diseases; c Student Research Committee, Faculty of Medicine; d Mazandaran University of Medical Sciences, Sari, Iran

**Keywords:** CIDP, COVID-19, GBS

## Abstract

**Introduction and importance:**

SARS-CoV-2 infection, besides respiratory symptoms, as cardinal manifestation, may present with neurological involvement. Immune-mediated polyradiculoneuropathy is one of the important neurological complications manifested by COVID-19 mainly includes Guillain–Barré syndrome (GBS), treatment-related fluctuation of GBS, and chronic inflammatory demyelinating polyradiculoneuropathy (CIDP). Also, there are several reports of COVID-19 vaccine-related GBS and CIDP. According to possible severe manifestations of neuropathies like respiratory failure, considering these complications for early diagnosis and treatment is very important.

**Case presentation:**

The authors presented a 67-year-old woman with severe weakness in upper and lower extremities, the patient was diagnosed as case with CIDP initiated after COVID-19 virus infection and/or vaccination that experienced one relapse in 11 months. In both episodes, treatment with intravenous immunoglobulin started and the patient respond.

**Clinical discussion:**

To the best of our knowledge, this is one of the first reported cases with a typical chronic course of CIDP associated with COVID-19 virus infection and/or vaccination.

**Conclusion:**

Considering this complication and differentiation between GBS and CIDP, and then initiating maintenance therapy can prevent long-term disability.

## Background

Highlights

- The COVID-19 was first described in late December 2019 in Wuhan, China.
- Polyradiculoneuropathy is one of the important neurological complications manifested by COVID-19 mainly includes Guillain–Barré syndrome, treatment-related fluctuation of Guillain–Barré syndrome, and chronic inflammatory demyelinating polyradiculoneuropathy.
- Chronic inflammatory demyelinating polyradiculoneuropathy is an autoimmune disease that participates in both humeral and cellular immune responses.

The COVID-19 was first described in late December 2019 in Wuhan, China^1^. The WHO reported about 590 million confirmed cases and 6.4 million deaths due to COVID-19^2^ . As well as flu-like symptoms and lung involvement, COVID-19 causes neurologic manifestations, including olfactory/taste disorders, myalgia, headache, altered mental status, and polyradiculoneuropathy^3^.

Polyneuropathy (or polyradiculoneuropathy) exhibits progressive muscle weakness, sensory impairment, and decreased or absent reflexes. Autoimmune processes can cause it against the peripheral nervous system. Immune-mediated neuropathies mainly consist of acute diseases, like Guillain–Barré syndrome (GBS) and its subtypes (axonal and demyelinating), or chronic diseases such as chronic inflammatory demyelinating polyradiculoneuropathy (CIDP)^4^. Like GBS, CIDP is characterized by progressive sensorimotor neuropathy but must be progressive for at least 8 weeks or more^5^.

Unlike the typical course of CIDP presentation as slowly progressive and/or relapsing over 8 weeks, 16% of patients present acutely resembling GBS. In these circumstances, the minimum meantime for acute-onset CIDP symptoms is typically more than 4 weeks despite less than 4 weeks for GBS. Patients with GBS may experience deterioration of symptoms after initiating treatment, referred to as treatment-related fluctuations (TRF). In these situations, acute-onset CIDP should be considered if this worsening occurs at least three times or after 8 weeks from the onset of the disease^6,7^. CIDP is an autoimmune disease that participates in both humeral and cellular immune responses. Nevertheless, the exact aetiology of this disease is unknown^8^. Here, we present a patient presented with CIDP after COVID-19 virus infection and vaccination. This case report has been reported in line with the SCARE Criteria^9^.

## Case presentation

**Figure 1:**
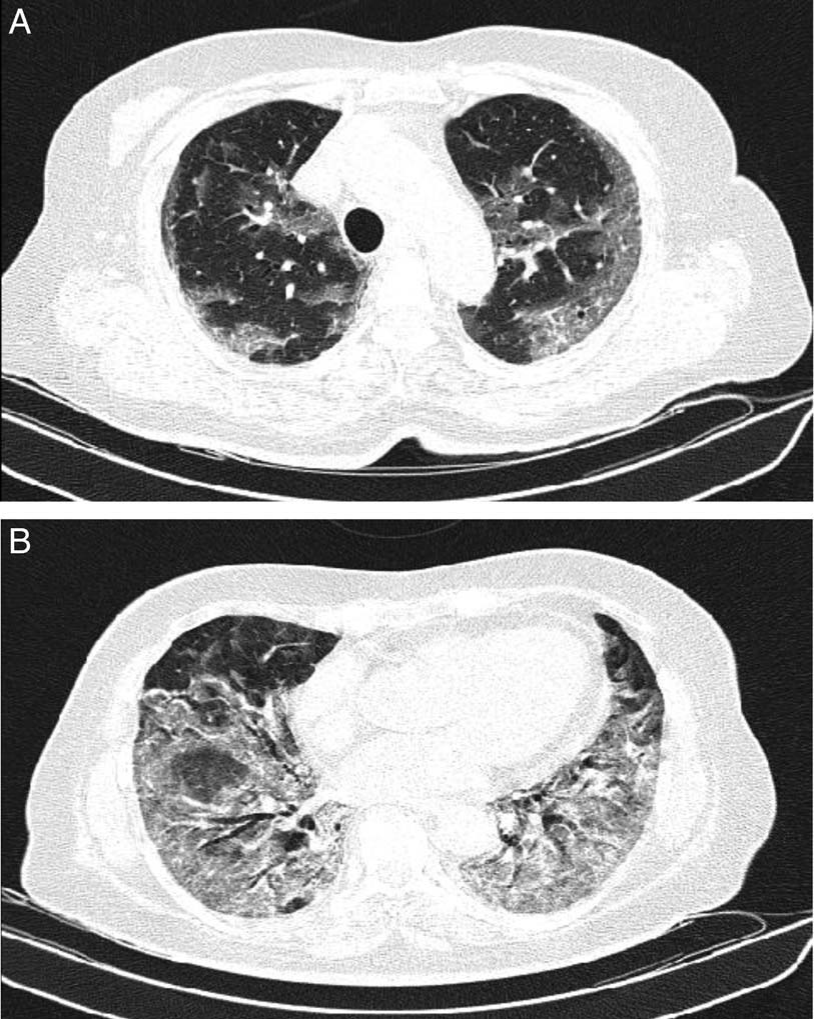
Two axial sections of the chest computed tomography scan: bilateral peripheral ground glass opacities in upper lobes (A) and diffuse consolidative and ground glass opacities in lower lobes (B).

In late July 2021, a 67-year-old woman was referred to another hospital with a complaint of fever, myalgia, mild dyspnoea, headache, and non-productive cough that started 4 days ago. She had no underlying diseases. No other complaint was noted. At first clinical evaluation in the emergency department, the blood pressure were 115/75 mmHg, a heart rate of 90 beats/min, a respiratory rate of 16 breaths per min, a temperature of 38.3°C, and oxygen saturation (SpO_2_) of 91%. In physical examination, no remarkable findings were have existed. SARS-CoV-2 polymerase chain reaction test from the upper respiratory tract was positive. Because of mild dyspnoea and decreased SpO_2_, a Chest computed tomography scan was performed. Peripheral consolidation and ground glass opacities were seen, as shown in Figure 1. Dexamethasone 8 mg/day for 10 days with Remdesivir 200 mg on the first day, then 100 mg/day for the next 4 days was initiated with supplemental oxygen. After 3 weeks, she was discharged while she had a mild weakness with normal vital signs (SpO_2_= 97%) and no significant findings in the physical examination.

After about 2 months, in late October, she was referred to the emergency department (ED) of the Razi hospital, Qaemshahr, northern Iran, with severe weakness in upper and lower extremities, which had begun gradually from prior hospitalization and exacerbated in these 2 months. She also complained of sensory disturbances. No other complaints were noted. Vital signs and SpO_2_ were normal. In physical examination, weakness grade 1 (according to Medical Research Council Scale for muscle strength)^10^ in upper extremity and grade 0 lower extremity, distal and proximal were found with the sensory deficit and areflexia in all limbs. Other organs were normal in physical exams. According to her clinical manifestations, we suspect immune-mediated polyneuropathies, especially after viral infection. We consulted with a neurologist, and an operating nerve conduction study and electromyography (EMG-NCV) were recommended. In the NCV study, conduction slowing velocity and axonal sensory and motor polyneuropathy was found that be consistent with CIDP according to established criteria^11^.

So, the patient was treated with Immunoglobulins, Intravenous (IVIG) 25 g daily (about 0.4 g per kg) for 5 days. After 1–2 weeks, symptoms improved, upper and lower extremities strength were 4–5, reflexes and sensory deficits recovered, and gradually she was able to walk with a two-wheel walker. The 25-g IVIG per month was repeated for the next 3 months and then stopped.

Six months later, she returned to the hospital with similar symptoms, motor and sensory deficits with hyporeflexia. Prior treatment with the 25-g IVIG daily for 5 days was repeated. Partial improvement after 1 week occurred, and the patient was discharged.

## Discussion and conclusion

As the COVID-19 pandemic emerged, a wide spectrum of clinical symptoms related to COVID-19 virus infection was reported from asymptomatic patients to severe respiratory involvement and extrapulmonary manifestations like a variety of neurological findings^3^. Although the exact mechanism is not fully known, probably neurologic involvement is caused by the virus itself because of the common human coronavirus’s neurotropic nature or by host immune response stimulation^12^. Polyneuropathy is one of several COVID-19 neurological findings. Immune-mediated polyradiculoneuropathies are diseases in which both humoral and cellular immune responses act against neurons by producing autoantibodies and T cells, respectively^13^. Here we presented a patient with symmetrical polyneuropathy of four limbs involvement after SARS-CoV-2 infection or vaccination.

Several cases of CIDP after COVID-19 vaccination and GBS after COVID-19 virus infection and vaccination have been reported. Sedaghat Z *et al.* ^14^ reported a patient who presented with symmetrical progressive distal polyneuropathy with a history of prior COVID-19 virus infection, who was treated by IVIG according to high suspicion of GBS. Albert P and colleagues reported that a 71-year-old man who manifested distal polyradiculoneuropathy progressed rapidly within 3 days, concomitant with COVID-19-related pneumonia from 1 week ago. By considering GBS as the most suspicious diagnosis, IVIG and other supportive care were initiated. But the patient did not respond and died due to progressive respiratory failure^15^. In another study, seven cases of GBS following the COVID-19 vaccine (ChAdOx1-S/nCoV-19) reported that all of them occurred in the first 2 weeks of receiving the first dose^16^

As reported in many studies, GBS can occur after infection or vaccination. But it seems many of them lack long-term follow-up, to distinguish whether they were GBS/TRF or CIDP with acute onset.

Bagella and colleagues reported a 49-year-old man, who presented with asymmetric bilateral facial weakness and paraesthesia in the tongue and face about 2 weeks after receiving the first dose of the ChAdOx1 nCoV-19 vaccine. No evidence of the previous infection was found. According to clinical course and paraclinical evaluations, GBS has been established and he received one cycle of 5-day IVIG, 0.4 g/kg/day. After 2 months symptoms relapsed, and EMG-NCV was done. Considering EMG-NCV and relapsing after 2 months, the diagnosis of CIDP was confirmed and he received the second course of IVIG. Later he was treated with IVIG cycles every 6 weeks and in 6 months follow-ups, symptoms improved and no relapses were found^17^.

In our study, symptoms began after severe pneumonia due to COVID-19 and vaccination (but with the inactive vaccine, Sinopharm). In addition, the clinical course of our patient was not acute but gradually. Treatment with IVIG tried, similar to our study, and clinical improvement with no relapse was established after maintenance therapy in this study, whereas we did not apply the maintenance dose of IVIG continuously after the first episode. This study showed, that it seems logical to continue IVIG periodically rather than stop that.

In another study, Suri and colleagues reported a 47-year-old man with a 2-year history of well-controlled diabetes mellitus and hypertension presented with rapidly progressive pure motor-flaccid quadriparesis with bilateral facial weakness with a history of COVID-19 pneumonia 7 months ago and recent receiving of the first dose of ChAdOx1 nCoV-19 vaccine, 17 days before this presentation. After two relapses in 8 weeks from the onset of first neurological symptoms despite receiving two courses of 0.4 g/kg body weight IVIG, daily for 5 days, the diagnosis of acute-relapsing CIDP has confirmed it may be occurred due to COVID-19 infection or COVID-19 vaccine. then managed by a 5-day course of IVIG and then maintenance therapy by 1 mg/kg/day of oral prednisolone and azathioprine at 100 mg/day which was different from our approach which and led to improvement of symptoms with no further relapses^7^. The Clinical scenario of this study was much similar to our patient’s especially the history of prior pneumonia and vaccination together, but our patient did not have diabetes mellitus as a risk factor for polyneuropathy^18^.

In our study, we had a patient with a history of severe pneumonia due to COVID-19 infection that presented with symmetrical sensorimotor polyradiculoneuropathy with areflexia without cranial nerve involvement, which began and deteriorate gradually within 2 months. No other previous illness except for the recent COVID-19 vaccination 1 week ago was noted^19^. A Clinical course of 8 weeks and EMG-NCV findings guide us toward chronic inflammatory demyelinating polyradiculoneuropathy, so 5 days of IVIG 0.4 g/kg/day was initiated followed by 0.4 mg/kg per month for 3 months continued, and then stopped. After 5 months she came back with a relapse of symptoms while receiving no maintenance therapy.

Our patient had one relapse of CIDP after 8 months after the first episode again treated by IVIG with the same prior dose. According to response to treatment as improvement of symptoms, our plan is maintenance therapy by 0.4 g/kg per dose every 4 weeks besides initiating rehabilitation.

As we know up to now, this is the first case of slowly progressive new-onset CIDP after COVID-19 virus infection and/or vaccination that has been reported. Long-term follow-up is needed to compare the response to treatment, risk of relapse, and long-term disability between using IVIG, corticosteroids, or immunosuppressive drugs (like azathioprine), alone, and a combination of those.

Further investigation needs to be done considering the polyneuropathies induced by human-coronaviruses, and whether the virus itself damages peripheral nerves or factors mediating inflammatory response, like vaccination, are responsible for the neural damage. Furthermore, the early diagnosis of the underlying cause, such as CIDP, would help us to begin the therapy immediately and therefore reduce secondary axonal degeneration induced by an immune response and long-term disability.

## Ethics approval

Considering Iran national committee for ethics in biomedical research lows. It’s not necessary to get ethical approval code for case reports, only patients consent is enough.

## Consent

Written informed consent was obtained from the patient for publication of this case report and accompanying images. A copy of the written consent is available for review by the Editor-in-Chief of this journal on request.

## Source of funding

None.

## Author contribution

M.-A.A. was involved in interpretation and collecting of data, and editing the manuscript. M.A.-S., L.-D., M.K. and S.K. were involved in writing, editing and preparing the final version of manuscript. All authors reviewed the paper and approved the final version of the manuscript.

## Conflicts of interest disclosure

The authors declare that no conflict of interest.

## Research registration unique identifying number (UIN)

1. Name of the registry:
2. Unique Identifying number or registration ID:
3. Hyperlink to your specific registration (must be publicly accessible and will be checked):

## Guarantor

Mohammad-Ali Abedini.

## Availability of data and materials

The datasets used and/or analyzed during the current study are available from the corresponding author on reasonable request.

## provenance and peer review

Not commissioned, externally peer-reviewed.

## References

[1] Mashamba-Thompson TP Crayton ED. Blockchain and artificial intelligence technology for novel coronavirus disease 2019 self-testing. MDPI. 2020 10:198. 10.3390/diagnostics10040198 PMC7235895 32244841

[2] WHO. Coronavirus disease 2022 WHO Corona virus disease. https://www.who.int/emergencies/diseases/novel-coronavirus-2019

[3] Tsai S-T Lu M-K San S. The neurologic manifestations of coronavirus disease 2019 pandemic: a systemic review. Front Neurol 2020;11:498. 32574246 10.3389/fneur.2020.00498 PMC7248254

[4] Kieseier BCME Sommer C Hartung HP. Immune-mediated neuropathies. Nat Rev Dis Prim 2018;1:1–23. 10.1038/s41572-018-0027-2 30310069

[5] Koski C Baumgarten M Magder L. Derivation and validation of diagnostic criteria for chronic inflammatory demyelinating polyneuropathy. J Neurol Sci 2009;277:1–8. 19091330 10.1016/j.jns.2008.11.015

[6] de Souza A Oo WM Giri P. Inflammatory demyelinating polyneuropathy after the ChAdOx1 nCoV-19 vaccine may follow a chronic course. J Neurol Sci 2022;436:120231. 35313224 10.1016/j.jns.2022.120231 PMC8923716

[7] Suri V Pandey S Singh J. Acute-onset chronic inflammatory demyelinating polyneuropathy after COVID-19 infection and subsequent ChAdOx1 nCoV-19 vaccination. BMJ Case Rep CP 2021;14:e245816. 10.1136/bcr-2021-245816 PMC8491284 34607818

[8] Eldar AH Chapman J. Guillain Barré syndrome and other immune mediated neuropathies: diagnosis and classification. Autoimmun Rev 2014;13:525–530. 24434363 10.1016/j.autrev.2014.01.033

[9] Agha RA Franchi T Sohrabi C. The SCARE 2020 guideline: updating consensus surgical CAse REport (SCARE) guidelines. Int J Surg 2020;84:226–30. 33181358 10.1016/j.ijsu.2020.10.034

[10] Taheri A Davoodi L Soleymani E. New‐onset myasthenia gravis after novel coronavirus 2019 infection. Respirol Case Rep 2022;10:e0978. 35620352 10.1002/rcr2.978 PMC9125167

[11] Van den Bergh PY van Doorn PA Hadden RD. European Academy of Neurology/Peripheral Nerve Society guideline on diagnosis and treatment of chronic inflammatory demyelinating polyradiculoneuropathy: Report of a joint Task Force—Second revision. J Peripheral Nerv Syst 2021;26:242–68. 10.1111/jns.12455 34085743

[12] Desforges M Le Coupanec A Dubeau P. Human coronaviruses and other respiratory viruses: underestimated opportunistic pathogens of the central nervous system? Viruses 2019;12:14. 31861926 10.3390/v12010014 PMC7020001

[13] Mathey EK Park SB Hughes RA. Chronic inflammatory demyelinating polyradiculoneuropathy: from pathology to phenotype. J Neurol Neurosurg Psychiatry 2015;86:973–985. 25677463 10.1136/jnnp-2014-309697 PMC4552934

[14] Sedaghat Z Karimi N. Guillain Barre syndrome associated with COVID-19 infection: a case report. J Clin Neurosci 2020;76:233–235. 32312628 10.1016/j.jocn.2020.04.062 PMC7158817

[15] Alberti PBS Piatti M Karantzoulis A. Guillain-Barré syndrome related to COVID-19 infection. Neurol Neuroimmunol Neuroinflam 2020;7:e741. 10.1212/NXI.0000000000000741 PMC7217652 32350026

[16] Maramattom BV Krishnan P Paul R. Guillain‐Barré syndrome following ChAdOx1‐S/nCoV‐19 vaccine. Ann Neurol 2021;90:312–314. 34114256 10.1002/ana.26143

[17] Bagella CF Corda DG Zara P. Chronic inflammatory demyelinating polyneuropathy after ChAdOx1 nCoV-19 vaccination. Vaccines 2021;9:1502. 34960248 10.3390/vaccines9121502 PMC8706382

[18] Román-Pintos LM Villegas-Rivera G Rodríguez-Carrizalez AD. Diabetic polyneuropathy in type 2 diabetes mellitus: inflammation, oxidative stress, and mitochondrial function. J Diabetes Res 2016;2016:3425617. 28058263 10.1155/2016/3425617 PMC5183791

[19] Koike H Chiba A Katsuno M. Emerging infection, vaccination, and guillain-barré syndrome: a review. Neurol Ther 2021;10:523–537. 34117994 10.1007/s40120-021-00261-4 PMC8196284

